# Supplementary material for: Carbon Monoxide-Loaded Red Blood Cell Prevents the Onset of Cisplatin-Induced Acute Kidney Injury
Source: Antioxidants (Basel). 2023 Sep 1;12(9):1705. doi: 10.3390/antiox12091705 (PMC10526101; doi:10.3390/antiox12091705)
Supplement: Supplementary file 1 [file antioxidants-12-01705-s001.zip › antioxidants-2544682-supplementary.pdf]

Supplemental Materials

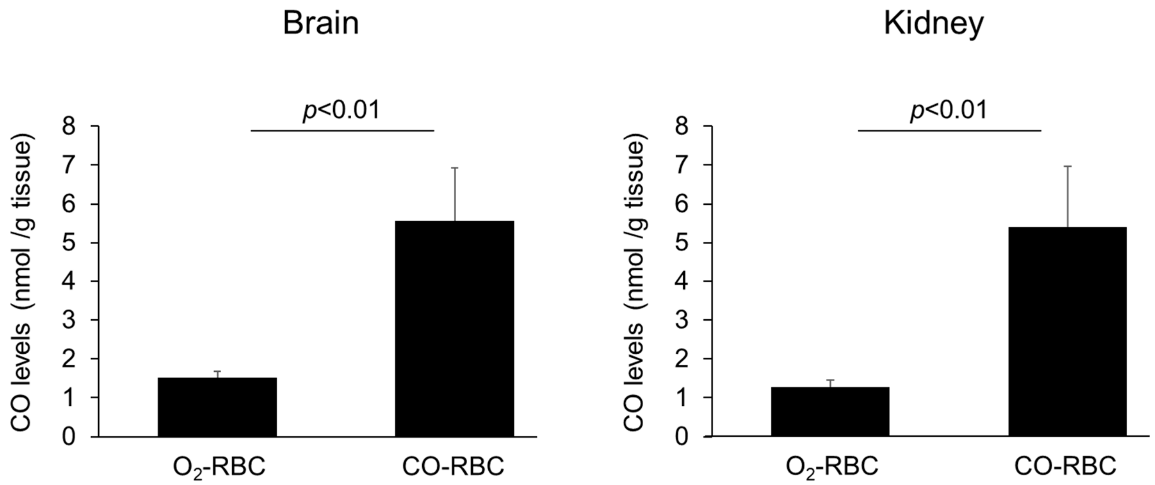

Figure S1. CO-RBC supplies CO to brain and kidney tissue. CO levels in whole brain or kidney of normal mice at 90 min after the administration of O_2-RBC (1400 mgHb/kg) and CO-RBC (1400 mgHb/kg) were measured by gas chromatography (n = 4/group). Results are expressed as the mean ± S.E.M.

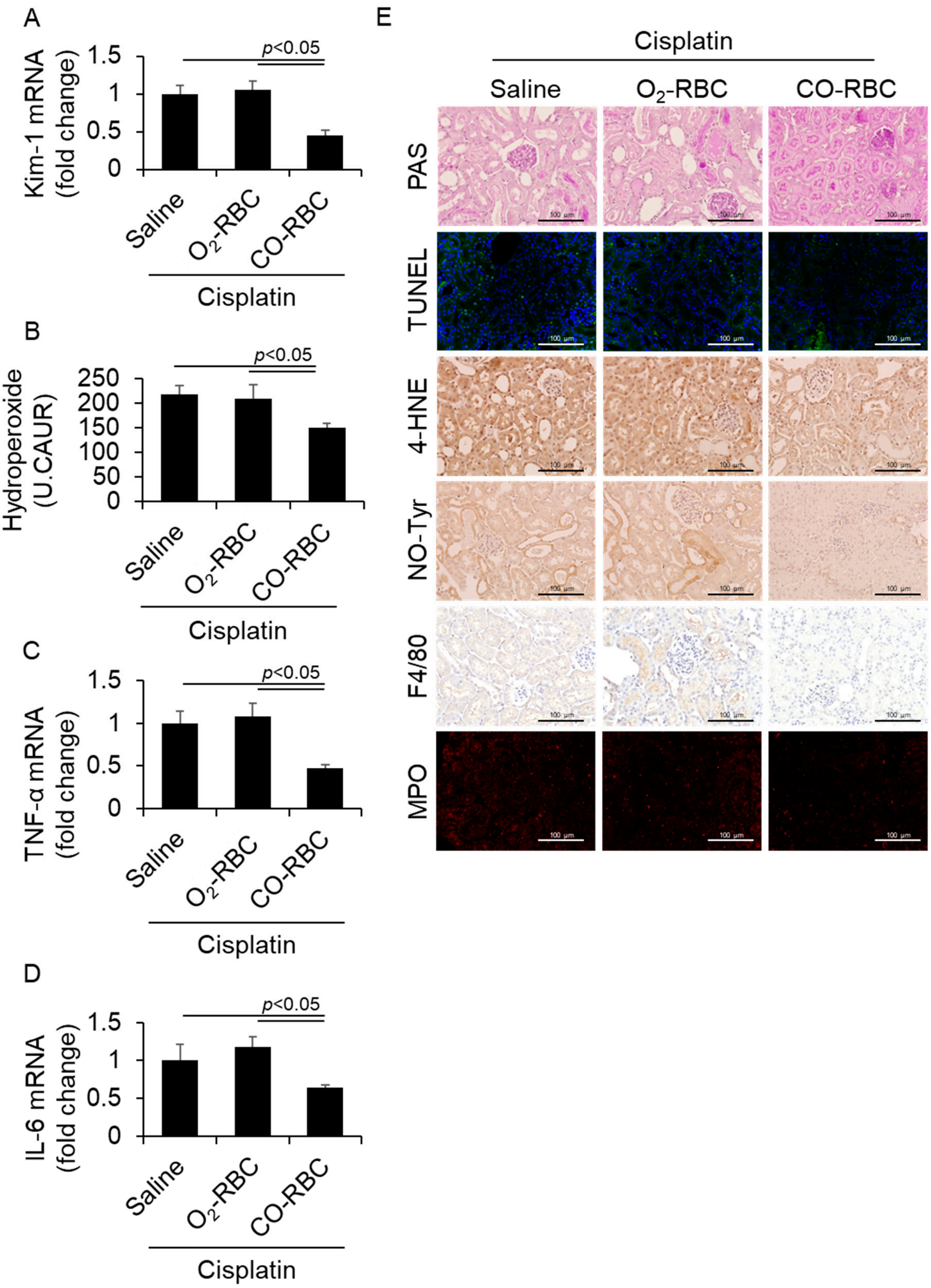

Figure S2. The therapeutic effects of O_2-RBC against cisplatin-induced AKI. To evaluate the effect of O_2-RBC on renal injury induced by the cisplatin treatment, kidneys were obtained

from cisplatin-induced AKI mice after the administration of saline (control), O_2-RBC (1400 mgHb/kg), or CO-RBC (1400 mgHb/kg). Tubular cell injury, oxidative stress, and inflammation were then analyzed by (A, C, D) qRT-PCR (n=4/group), (B) d-ROMs test (n=4/group), and (E) PAS staining, TUNEL staining, and immunostaining with antibodies against 4-HNE, NO-Tyr, F4/80, and MPO (magnification, ×400, scale bars, 100 μm). Results are expressed as the mean ± S.E.M.

Table S1. List of primers used in this study.

Target gene (Accession number)	Forward (5'-3')	Reverse (5'-3')
Human Kim-1 (NM_001173393.3)	GACAACGAGCATTCCAACAA	GTCACAGTGCCATTCCAGTC
Mouse Kim-1 (NM_001166631.1)	TCCACACATGTACCAACATCAA	GTCACAGTGCCATTCCAGTC
Human TNF-α (NM_000594.4)	GGCTCCAGGCGGTGCTTGTT	GGCGGTTCAGCCACTGGAGC
Mouse TNF-α (NM_001278601.1)	TTCAGAGCCGTTGGTGTATC	CCCATTCCAGGTAGGTGTTT
Human IL-6 (NM_000600.5)	CAGTTCCTGCAGAAAAAGGC	AACAACAATCTGAGGTGCCC
Mouse IL-6 (NM_001314054.1)	TCTCTGCAAGAGACTTCCATCC	AGACAGGTCTGTTGGGAGTG
Human GAPDH (NM_001256799.3)	GGTGAAGGTCGGAGTCAACG	ACCATGTAGTTGAGGTCAATGAAGG
Mouse GAPDH (NM_001289726.2)	AACTTTGGCATTGTGGAAGG	ACACATTGGGGGTAGGAACA
